# Developing Technology for Nursing Home Implementation: An Engineering and Social Science Collaboration

**DOI:** 10.1093/geroni/igaf122.2061

**Published:** 2025-12-31

**Authors:** A Lynn Snow, Todd Freeborn, Kimberly Lim, Brian Cox, Vanessa Aguilar, Hope Carwile

**Affiliations:** The University of Alabama, Tuscaloosa, Alabama, United States; The University of Alabama, Tuscaloosa, Alabama, United States; The University of Alabama, Tuscaloosa, Alabama, United States; Alabama Research Institute on Aging, Tuscaloosa, Alabama, United States; The University of Alabama, Tuscaloosa, Alabama, United States; Vivage, Lakewood, Colorado, United States

## Abstract

Although temperature, light, and noise are known to be key factors for a good night’s sleep, they are difficult to measure in the nursing home. We report on a multidisciplinary collaboration (engineering, psychology, nursing, social work) to develop a flexible, unobtrusive, and affordable a measurement solution feasible for nursing home use. We piloted commercially available instruments that measured volume and light in two nursing homes. We trialed different solutions for powering these devices, downloading the data, and placing the devices in resident rooms and common areas. We concluded that commercially available instruments were too large, noticeable, and expensive. We found that: 1) large noticeable microphones led to privacy concerns even though the devices were only measuring sound levels and were not recording; 2) strategic and customized device placement was key to success – in some resident rooms devices did best when placed above doors, and in other resident rooms the device could be successfully placed on a piece of furniture like a chest of drawers; 3) connecting to nursing home wifi was not typically possible, so data download solutions that relied on removable memory cards worked best. Based on pilot work, the second and third authors developed a small bespoke device on a raspberry pi platform that is wall mountable with heavy-duty Velcro and measures sound, temperature, and light using removable SD memory cards and has both battery and AC power options. The device is being used to supplement wrist actigraph/light meters. We report on nursing home feasibility and acceptability.

